# Digital Literacy and Dementia Knowledge Among Older Asian Americans

**DOI:** 10.1093/geroni/igaf122.2851

**Published:** 2025-12-31

**Authors:** Nan Sook Park, Yuri Jang, Hongdao Meng, Soonhyung Kwon, Soondool Chung, David A Chiriboga

**Affiliations:** University of South Florida, Tampa, Florida, United States; University of Southern California, Los Angeles, California, United States; University of South Florida, Tampa, Florida, United States; University of South Florida, Tampa, Florida, United States; Ewha Womans University, Seoul, Republic of Korea; University of South Florida, Tampa, Florida, United States

## Abstract

Dementia knowledge is essential for early detection, care planning, and reducing stigma. Disparities persist among immigrant and minority populations, especially among older Asian Americans. Grounded in the digital health literacy model, this study examined how immigration and social factors, digital literacy, and dementia-related variables would be associated with dementia knowledge. Data were drawn from a survey with 507 Asian Americans aged 50 and older (M = 64.42, SD = 9.68) in central Florida. Hierarchical regression analyses estimated the impact of sequential blocks of independent variables on dementia knowledge: (1) background characteristics (age, gender, education, financial status, chronic conditions), (2) immigration-related factors (limited English proficiency [LEP], length of U.S. residence), (3) social factors (marital status, social network, activity participation), (4) digital literacy, health-information seeking behavior, and confidence in health information, and (5) dementia-related factors (having a family/friend with dementia, concerns about dementia, concerns about caregiving, dementia planning). Digital literacy emerged as a major independent predictor of dementia knowledge (β = .22, p < .001). Being married, having a family member/friend with dementia, and perceived importance of dementia planning as well as female gender and shorter US residence were also significant knowledge promoters. LEP was negatively associated with dementia knowledge, but its effect diminished when digital literacy was included. Findings suggest the critical role of digital literacy in reducing dementia knowledge disparities. Interventions enhancing digital and eHealth literacy, particularly among older adults with LEP, can improve access to dementia-related information. Future efforts should develop culturally tailored digital resources to bridge knowledge gaps.

